# Synthesis, Structure, and Antiproliferative Activity of Three Gallium(III) Azole Complexes

**DOI:** 10.1155/2010/168030

**Published:** 2010-07-18

**Authors:** Stergios Zanias, Giannis S. Papaefstathiou, Catherine P. Raptopoulou, Konstantinos T. Papazisis, Vasiliki Vala, Dimitra Zambouli, Alexandros H. Kortsaris, Dimitrios A. Kyriakidis, Theodoros F. Zafiropoulos

**Affiliations:** ^1^Department of Chemistry, University of Patras, 265 04 Patras, Greece; ^2^Laboratory of Inorganic Chemistry, Department of Chemistry, National and Kapodistrian University of Athens, Panepistimiopolis, 157 71 Zografou, Greece; ^3^Institute of Materials Science, NCSR “Demokritos”, 153 10 Aghia Paraskevi Attikis, Greece; ^4^“Theagenion” Cancer Hospital, Al. Simeonides str. 2, 540 07 Thessaloniki, Greece; ^5^Department of Chemistry, Aristotle University of Thessaloniki, 541 24 Thessaloniki, Greece; ^6^The National Hellenic Research Foundation, 48, Vas. Constantinou Ave, 116 35 Athens, Greece

## Abstract

As part of our interest into the bioinorganic chemistry of gallium, gallium(III) complexes of the azole ligands 2,1,3-benzothiadiazole (btd), 1,2,3-benzotriazole (btaH), and 1-methyl-4,5-diphenylimidazole (L) have been isolated. Reaction of btaH or btd with GaBr_3_ or GaCl_3_ resulted in the mononuclear complexes [GaBr_3_(btaH)_2_] (**1**) and [GaCl_3_(btd)_2_] (**2**), respectively, while treatment of GaCl_3_ with L resulted in the anionic complex (LH)_2_[GaCl_4_] (**3**). All three complexes were characterized by single-crystal X-ray crystallography and IR spectroscopy, while their antiproliferative activities were investigated against a series of human and mouse cancer cell lines.

## 1. Introduction

The coordination chemistry of gallium(III) has become an area of increasing research activity due to its relevance with both materials science [1–6] and biomedical developments [7–21]. In the area of materials science, for example, complex [Ga_2_(saph)_2_q_2_], where saph^2−^ is the Schiff-base ligand bis(salicylidene-o-aminophenolate)(-2) and q^−^ is 8-quinolinate(-1), is a very good candidate as a novel electron-transporting and emitting material for organic light-emitting diodes (OLEDs) [4]. [Gaq_3_] is also a promising electroluminescence (EL) material, exhibiting higher power efficiency than the aluminum analogue, [Alq_3_] [5, 6]. The biological interest of gallium(III) complexes originates from the incorporation of gallium(III) radionuclides (^67^Ga^3+^, ^68^Ga^3+^) into diagnostic radiopharmaceuticals [7]. In addition, the gallium salts GaCl_3_ and Ga(NO_3_)_3_ as well as few gallium(III) complexes [8–19] have exhibited antitumour activity, while Ga(NO_3_)_3_ and some GaCl_3_/L complexes (L = various azoles) showed in vitro anti-HIV (HIV = human immunodeficiency virus) activity [20]. The biological activity of gallium(III) complexes has often been attributed to the fact that gallium(III) is the diamagnetic biological mimic of iron(III) [21]. It is worth mentioning that [Gaq_3_], which is of current interest in materials science [5, 6], is also being evaluated in clinical trials, along with other Ga(III) complexes, such as gallium maltolate [tris(3-hydroxy-2-methyl-4H-pyran-4-onato)gallium(III)], for anticancer activity [22–24].

Following our interest in the coordination chemistry of gallium(III) [25–31] which is focused on the synthesis, structural characterization, physical/spectroscopic study and evaluation of the biological (antitumour and antiviral) activity of Ga(III) complexes with biologically relevant and nonrelevant ligands, we report herein the synthesis, structural characterization, and antiproliferative activity of three gallium complexes based on the azole ligands 2,1,3-benzothiadiazole (btd), 1,2,3-benzotriazole (btaH), and 1-methyl-4,5-diphenylimidazole (L).

## 2. Experimental

### 2.1. Reagents and Physical Measurements

All manipulations were performed under a dinitrogen atmosphere, using standard inert atmosphere techniques and purified solvents unless otherwise noted. All other chemicals were purchased from commercial sources and used without further purification. L was synthesized as described elsewhere [32]. Microanalyses (C, H, and N) were performed by the University of Ioannina Microanalytical Laboratory using an EA 1108 Carlo Erba analyzer. IR spectra (4000–450 cm^−1^) were recorded on a Perkin-Elmer 16 PC spectrometer with samples prepared as KBr pellets. Far-IR spectra (500–50 cm^−1^) were recorded on a Bruker IFS 113v FT spectrometer as polyethylene pellets.

### 2.2. Compound Preparation

#### 2.2.1. Preparation of [GaBr_3_(btaH)_2_] (**1**) 

A solution of GaBr_3_ (0.3 g, 0.9 mmol) in 3 ml of toluene/diethyl ether (80 : 20, v/v) was added dropwise to a stirred solution of btaH (0.3 g, 2.5 mmol) in toluene (20 ml). The resultant solution was refluxed for about 3 hours and then left undisturbed at room temperature. Upon standing, X-ray quality colorless crystals of **1** formed over a period of 3 days. The crystals were collected by filtration, washed with toluene and dried in vacuum. Yield: 0.31 g (63%); *Anal. *Calc. for C_12_H_10_N_6_Br_3_Ga: C, 26.32; H, 1.84; N, 15.34. Found: C, 26.28; H, 1.82; N, 15.33%. Selected IR data (cm^−1^): 3238 m [*ν*(N–H)], 1222 mb [*ν*(N=N)], 1116 s [*ν*(N–N)], 291s [*ν*(Ga–Br)], and 224w [*ν*(Ga–N)].

#### 2.2.2. Preparation of [GaCl_3_(btd)_2_] (**2**)

A solution of GaCl_3_ (0.25 g, 1.40 mmol) in 5 ml of toluene/diethyl ether (80 : 20, v/v) was added dropwise to a stirred solution of btd (0.6 g, 4.4 mmol) in toluene/diethyl ether (60 : 40, v/v) (10 ml). The resultant solution was refluxed for about 2 hours and then left undisturbed at −10°C. Upon standing at low temperature for several days, X-ray quality yellowish crystals of **2** formed. The crystals were collected by filtration, washed with diethyl ether and dried in vacuum. Yield: 0.60 g (95%); m.p.: 112°C. *Anal. *Calc. for C_12_H_8_N_4_S_2_Cl_3_Ga: C, 32.14; H, 1.80; N, 12.49. Found: C, 32.13; H, 1.78; N, 12.49%. Selected IR data (cm^−1^): 1612 s and 1528 s [*ν*(C=C)], 1482 s [*ν*(C=N)], 961 m and 922 s [*ν*(S–N)], 382s [*ν*(Ga–Cl)], and 207 w [*ν*(Ga–N)].

#### 2.2.3. Preparation of (LH)_2_[GaCl_4_]Cl (**3**) 

A solution of GaCl_3_ (0.2 g, 1.13 mmol) in 5 ml of toluene/diethyl ether (80 : 20, v/v) was added dropwise to a stirred mixture of L (0.6 g, 2.6 mmol) in diethyl ether (1 ml). The resultant mixture was stirred until a clear yellowish solution was obtained. Slow evaporation of the resultant solution afforded a microcrystalline solid. The solid was collected by filtration, washed with toluene and diethyl ether, and dried in vacuum. The product was recrystallised three times from toluene to give crystals of **3** suitable for X-ray structural analysis. The crystals were collected by filtration, washed with toluene and dried in vacuum. Yield: 0.18 g (45%); *Anal. *Calc. for C_32_H_30_N_4_Cl_5_Ga: C, 53.56; H, 4.21; N, 7.81. Found: C, 53.36; H, 4.17; N, 7.78%. Selected IR data (cm^−1^): 3146–2620 sb [*ν*(N–H)], 1622 m [*ν*(C=N)], 1578 w [*ν*(C=C)], and 369s [*ν*(Ga–Cl)].

### 2.3. Single-Crystal X-Ray Crystallography

Crystals of **1** and **2** were mounted in air, while crystals of **3** were mounted in air and covered with epoxy glue. Diffraction measurements for **1** and **2** were made on a Crystal Logic Dual Goniometer diffractometer using graphite-monochromated Mo radiation, while those for **3** were made on a P21 Nicolet diffractometer using graphite-monochromated Cu radiation. Complete crystal data and parameters for data collection and processing are reported in Table 1. Unit cell dimensions were determined and refined by using the angular settings of 25 automatically centred reflections in the ranges 11 < 2*θ* < 23° for **1** and **2** and 22 < 2*θ* < 54° for **3**. Three standard reflections monitoring every 97 reflections showed less than 3% variation and no decay. Lorentz, polarization and *ψ*-scan (only for **1**) corrections were applied using CRYSTAL LOGIC software. The structures were solved by direct methods using SHELXS-86 [33] and refined by full-matrix least squares techniques on *F* ^2^ with SHELXL-97 [34]. All hydrogen atoms were located by difference maps and refined isotropically, except those on the methyl groups of **3** which were introduced at calculated positions as riding on bonded atoms. For all the three structures, all nonhydrogen atoms were refined using anisotropic thermal parameters.

### 2.4. In Vitro Cytotoxic Activity

#### 2.4.1. Test Substances

All test substances (complexes **1**, **2,** and **3**) were diluted in methanol at a concentration of 200 mM. Final concentration of methanol in culture was always less than 0.5%, a concentration that produced no effects on cell growth and proliferation, as was experimentally confirmed.

#### 2.4.2. Cell Lines

Cell lines used were HeLa [35] (human cervical cancer), OAW-42 [36] (human ovarian cancer), HT29 [37] (human colon cancer), MCF-7 [38] (human breast cancer), T47D [39] (human breast cancer), and L929 (929 is a clone isolated [40] from the parental strain L derived from normal subcutaneous areolar and adipose tissues of a mouse [41]). Cells were grown as monolayer cultures in T-75 flasks (Costar), were subcultured twice a week at 37°C in an atmosphere containing 5% CO_2_ in air and 100% relative humidity. Culture medium used was Dulbecco's modified Eagle's medium (DMEM, Gibco Glasgow, UK), supplemented with 10% Fetal Bovine Serum (FBS, Gibco, Glasgow, UK), 100 *μ*g/ml streptomycin and 100 IU/ml penicillin.

#### 2.4.3. Cell Growth and Proliferation Assays

Adherent cells at a logarithmic growth phase were detached by addition of 2-3 ml of a 0.05% trypsin (Gibco, 1 : 250) −0.02% EDTA mixture and incubation for 2–5 min at 37°C. Cells were plated (100 *μ*l per well) in 96-well flat-bottom microtiter plates (Costar-Corning, Cambridge) at a density of 5,000 (HeLa and L929) or 10,000 (HT-29, OAW-42, MCF-7 and T47D) cells per well. Cells were left for 24 h at 37°C to resume exponential growth. An equal volume (100 *μ*l) of either complete culture medium (control wells), or twice the final substance concentration diluted in complete culture medium, was added 24 h later. Six replicate wells for each concentration were used for the sulforhodamine B (SRB) assay and three replicate wells for the bromodeoxyuridine (BrdU) assay. Background control wells (*n* = 8), containing the same volume of complete culture medium, were included in each experiment. Cell growth or DNA-synthesis was evaluated 48 h later by means of the SRB or BrdU assays. All experiments were performed at least twice.

#### 2.4.4. SRB Assay

The SRB assay was carried out by a modification [42] of the previously reported method [43]. In brief, culture medium was aspirated prior to fixation using a microplate-multiwash device (Tri-Continent Scientific, Inc. Grass Valley, CA) and 50 *μ*l of 10% cold (4°C) TCA were gently added to the wells. Microplates were left for 30 min at 4°C, washed 5 times with deionized water and left to dry at room temperature for at least 24 hr. Subsequently, 70 *μ*l 0.4% (w/v) sulforhodamine B (Sigma) in 1% acetic acid solution were added to each well and left at room temperature for 20 min. SRB was removed and the plates were washed 5 times with 1% acetic acid before air drying. Bound SRB was solubilized with 200 *μ*l 10 mM unbuffered Tris-base solution (E. Merck, Darmstadt, Germany) and plates were left on a plate shaker for at least 10 min. Absorbance was read in a 96-well plate reader (Anthos-2001, Anthos labteck instruments, A-5022, Salzburg) at 492 nm subtracting the background measurement at 620 nm. The test optical density (OD) value was defined as the absorbance of each individual well, minus the blank value (“blank” is the mean optical density of the background control wells, *n* = 8). Mean values and CV from six replicate wells were calculated automatically. Results were expressed as the “survival fraction” (sf), derived from the following equation: sf = ODx/ODc, (where ODx and ODc represent the test and the control optical density, resp.). 

#### 2.4.5. BrdU Assay

DNA-synthesis was estimated by the BrdU assay [44] using a standard colorimetric ELISA (Boehringer Mannheim). After 47 h exposure to test substances, cells were incubated at 37°C for further 60 min in the presence of 10 *μ*M BrdU. Subsequently, cells were fixed with an ethanol-containing fixative, an anti-BrdU mouse monoclonal antibody conjugated with peroxidase was added and plates were incubated at 37°C for 60 min. After washing, peroxidase substrate (tetramethylbenzidine) was added, the reaction was stopped 10 min later by 1 M H_2_SO_4_ and absorbance was read at 450 nm subtracting the background measurement at 620 nm. Results from each triplicate well (ODBrdUx/ODBrdUc) were divided by the results of a parallel experiment estimated with the SRB assay (ODSRBx/ODSRBc) and they were expressed as the "DNA synthesis fraction" (fDNA) (derived from the following equation: fDNA = (ODBrdUx×ODSRBc)/(ODBrdUc×ODSRBx), where ODx and ODc represent the test and the control optical density resp.), resulting in an estimation of the DNA synthesis per cell number.

#### 2.4.6. Cell Cycle Analysis by Flow Cytometry

For cell cycle experiments 1.5 × 10^6^ (HeLa and L929) or 2.5 × 10^6^ (HT-29, OAW-42, MCF-7 and T47D) cells were seeded in 75 cm^2^ flasks and left for 24 h in incubator to resume exponential growth. Cells were exposed to test substances (at concentrations that produced 50% inhibition of cell growth—estimated by the SRB assay) and after 48 h they were harvested (using trypsin/EDTA as above), washed in PBS and counted in a hemocytometer chamber; 3×10^6^ cells were resuspended in 125 *μ*l cold “Saline GM” (g/L: glucose 1.1; NaCl 8.0; KCl 0.4; Na_2_HPO_4_·12H_2_O 0.39; KH_2_PO_4 _0.15; and 0.5 mM EDTA) followed by the addition of 375 *μ*l of 95% nondenatured, ice-cold ethanol [45]. Cells were kept in 4°C for a maximum period of 3 days (short-term storage does not alter results, as was experimentally confirmed) until analysis was performed.

For cell cycle analysis a 10% of standard chicken erythrocyte nuclei were added as a control. The samples were processed in a DNA-preparation Epics Workstation (Coulter, El). By this method the content of cellular DNA is assessed using Propidium Iodide [46, 47]. To avoid an increased signal by staining artifact on double stranded RNA, cells were digested with DNase-free RNase A [48].

Cellular DNA content was measured using an Epics II flow cytometer (Coulter, El). The fluorescent signals from 10,000–20,000 cells were collected and the result was displayed as a frequency-distribution histogram (DNA histogram). The mean channel, cell count, standard deviation (SD), coefficient of variation (CV), DNA index (DI), and cell cycle distribution were calculated for each sample using the Multicycle Cell Cycle Analysis Software (Phoenix Flow Systems Inc.). Care was taken to exclude any doublets or cell debris noise from the assessment.

## 3. Results and Discussion

### 3.1. Brief Synthetic Comments

Complexes **1** and **2** were prepared by the simple reactions of GaBr_3_ or GaCl_3_ and btaH or btd in toluene/diethyl ether under nitrogen employing 1 : 3 molar ratios, respectively. A similar reaction involving GaCl_3_ and btaH has yielded [GaCl_3_(btaH)_2_] [25]. An 1 : 1 complex of GaCl_3_/btaH has also been isolated and structurally characterized [25]. An attempt to isolate the 1 : 1 GaBr_3_/btaH complex was unsuccessful resulting in **1** in a lower yield. Complex **2** is also the only product resulting from the GaCl_3_/btd reaction mixtures in various molar ratios. Complex **3** might be regarded as a product of hydrolysis which is pretty usual in Ga(III) chemistry in water or water containing solutions [30].

### 3.2. IR Spectra

The IR spectrum of **1** exhibits a medium intensity band at ~3238 cm^−1^, assignable to *ν*(N–H). The bands at 1222 and 1116 cm^−1^ are attributed to the *ν*(N=N) and *ν*(N–N) vibrations, respectively, and are shifted to higher wavenumbers with respect to the spectrum of the free ligand (1208 versus and 1084 m, resp.). The IR spectrum of **2** exhibits three strong intensity bands at 1612, 1528 and 1482 cm^−1^ assignable to stretching carbon-carbon and carbon-nitrogen vibrations. These bands are not shifted significantly with respect to the spectrum of the free ligand [1608 w, 1518 s and 1476 s]. The bands at 950 and 916 cm^−1^ in the spectrum of btd, which are assigned to the *ν*(S–N) mode, have been shifted to higher wavenumbers in the spectrum of **2** [961 and 922 cm^−1^]. A set of broad bands in the region of 3146–2620 cm^−1^ in the spectrum of **3** can be assigned to the *ν*(N–H) of the protonated ligand, LH^+^. The *ν*(C=N) and *ν*(C=C) of the free L at 1602 and 1575 cm^−1^ have shifted to 1622 and 1578 cm^−1^ in the spectrum of **3** due to protonation.

The far-IR spectra of all three complexes are expected to show one Ga–X (X = Cl or Br) stretching mode [25] and these modes appear at 291s [*ν*(Ga–Br) in **1**], 382s [*ν*(Ga–Cl) in **2**], and 369s [*ν*(Ga–Cl) in **3**]. The far-IR spectra of complexes **1** and **2** exhibit one more band at 224 and 207 cm^−1^, respectively, which are attributed to the *ν*(Ga–N) mode [25].

### 3.3. Description of Structures

**Figure 1:**
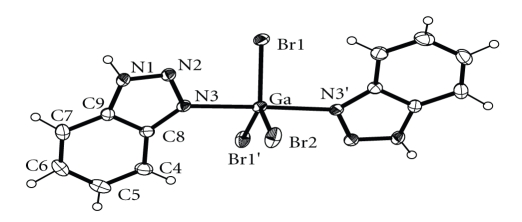
A labeled ORTEP plot of [GaBr_3_(btaH)_2_] (**1**) showing 30% probability ellipsoids.

**Figure 2:**
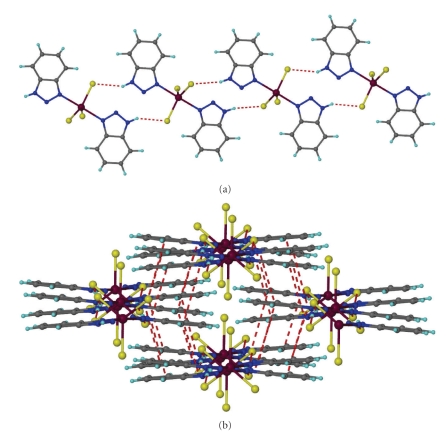
The hydrogen-bonded tape of [GaBr_3_(btaH)_2_] (**1**) running parallel to *a* axis (a) and the stacking of the tapes (b).

An ORTEP diagram of **1** is shown in Figure 1. Selected bond distances and angles are given in Table 2. Complex **1** is isostructural with [GaCl_3_(btaH)_2_] [25]. Its structure consists of the monomeric discrete [GaBr_3_(btaH)_2_] units. The gallium coordination geometry is trigonalbipyramidal with the bromo ligands defining the equatorial plane. There is a two-fold crystallographic axis along the Ga–Br2 bond. The Ga–N bond length in complex **1** [2.212(3) Å] is longer than that of [GaCl_3_(btaH)_2_] [2.169(2) Å]. The dihedral angle between the best planes of the btaH molecules is 10.90 Å and is larger than that of [GaCl_3_(btaH)_2_] [7.4°]. The N1 proton is hydrogen bonded to atom Br1 of a neighboring molecule [N1⋯Br1′ (1 − *x*, −*y*, 1 − *z*) 3.425(4) Å, HN1⋯Br1′ 2.64(7) Å and N1–HN1⋯Br1′ 149(6)°] creating a hydrogen-bonded tape running parallel to the *a* axis (Figure 2). These tapes are hold together in the crystal lattice through *π*-*π* interactions. Those interactions form between the phenyl groups of the coordinated btaH molecules of neighboring tapes [centroid⋯centroid′ (1 − *x*, 0.5 + *y*, 1.5 − *z*) 3.658(4) and 3.906(4) Å] (Figure 2).

**Figure 3:**
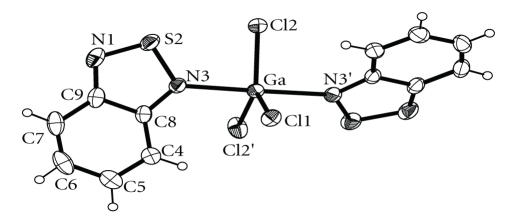
A labeled ORTEP plot of [GaCl_3_(btd)_2_] (**2**) showing 30% probability ellipsoids.

**Figure 4:**
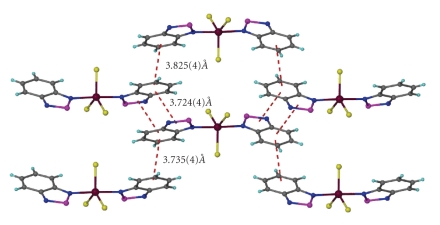
The stacking of the [GaCl_3_(btd)_2_] molecules in **2**.

Complex **2** crystallizes in the monoclinic space group *C*2/c. An ORTEP diagram of **2** is shown in Figure 3, while selected bond distances and angles are listed in Table 3. Its structure consists of monomeric discrete [GaCl_3_(btd)_2_] units. The gallium coordination geometry is again trigonalbipyramidal with the choro ligands defining the equatorial plane. There is a two-fold crystallographic axis along the Ga–Cl1 bond. The Ga–Cl bond lengths in complex **2** [2.171(2) and 2.180(1) Å] compare favourably with those of [GaCl_3_(btaH)_2_] [2.204(1) and 2.178(2) Å]. The Ga–N bond length in complex **2** [2.201(3) Å] is longer than that of [GaCl_3_(btaH)_2_] [2.169(2) Å], but compares well with that of **1** [2.212(3) Å]. The dihedral angle between the best planes of the btd molecules is 52.51 Å and is much larger than that of **1** and [GaCl_3_(btaH)_2_] (10.90 and 7.4°, resp.). There appear to be intermolecular stacking interactions between the nearly parallel btd ligands. Those interactions involve both the thiadiazole and the phenyl groups of the btd ligands as shown in Figure 4.

**Figure 5:**
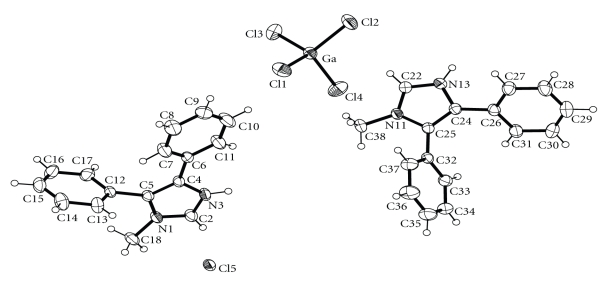
A labeled ORTEP plot of the asymmetric unit of (LH)_2_[GaCl_4_]Cl (**3**), showing 30% probability ellipsoids.

**Figure 6:**
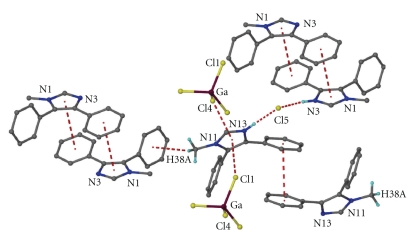
The intermixture of the weak interactions between the anions and the cations in **3**. Most of the hydrogen atoms have been omitted for clarity.

An ORTEP diagram of the asymmetric unit of **3** is shown in Figure 5. Selected bond distances and angles are listed in Table 4. The crystal of **3** consists of protonated LH^+^ ligand cations, tetrachlorogallate(III) anions and Cl^−^ anions. The Ga–Cl distances in the tetrahedral [GaCl_4_]^−^ ion are in the narrow range 2.152(1)–2.173(1) A° with the Cl–Ga–Cl angles varying from 107.1(1)° to 110.9(1)°. These values are similar to those observed for other complexes containing the tetrachlorogallate(-1) ion [13, 29]. The crystal structure of (LH)_2_[GaCl_4_]Cl is dominated by an intermixture of N–H⋯Cl and C_Me_–H⋯*π* hydrogen bonds, Ga–Cl⋯*π*_azole_ and *π*-*π* interactions (Figure 6). The organic moieties LH^+^ are connected through N–H⋯Cl and C–H⋯*π*_phenyl_ interactions to form a chain; data are as follows N3⋯Cl5′ (2 − *x*, −*y*, 2 − *z*) 3.088(3) Å, HN3⋯Cl5′ 2.24(4) Å and N3–HN3⋯Cl5′ 160(4)°; N13⋯Cl5′′ (*x* − 1, 1 + *y*, *z* − 1) 3.066(4) Å, HN13⋯Cl5′′ 2.19(5) Å and N13–HN13⋯Cl5′′ 177(5)°; C38⋯Centroid′ (1 − *x*, −*y*, 2 − *z*) 3.691(5) Å, H38A⋯Centroid′ 2.85(1) Å and C38–H38A⋯Centroid′ 147(1)°. The organic chains are bridged through Ga–Cl⋯*π*_azole_ interactions to form layers [Cl1⋯Centroid′′ (−*x*, 1 − *y*, 1 − *z*) 3.455(2) Å and Cl4⋯Centroid′′′ (1 − *x*, 1 − *y*, 1 − *z*) 3.550(2) Å], which are further bridged through *π*-*π* interactions in the third dimension [centroid⋯centroid′ (1 − *x*, −*y*, 2 − *z*) 3.778(3) and centroid⋯centroid′′′′ (−*x*, 2 − *y*, 1 − *z*) 3.878(3)].

### 3.4. Antiproliferative Activity

**Figure 7:**
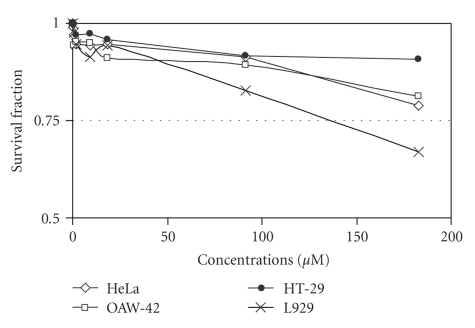
Dose-effect plots of complex **1 **against a panel of human and mouse cancer cell lines 24 h after the administration of the agents. Cytotoxicity was estimated via SRB assay (each point represents a mean of six replicate wells).

**Figure 8:**
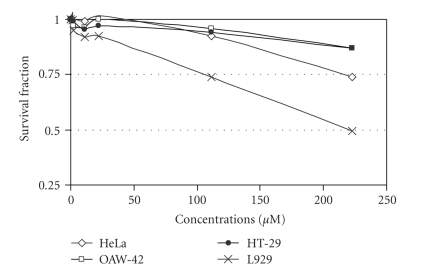
Dose-effect plots of complex **2 **against a panel of human and mouse cancer cell lines 24 h after the administration of the agents. Cytotoxicity was estimated via SRB assay (each point represents a mean of six replicate wells).

**Figure 9:**
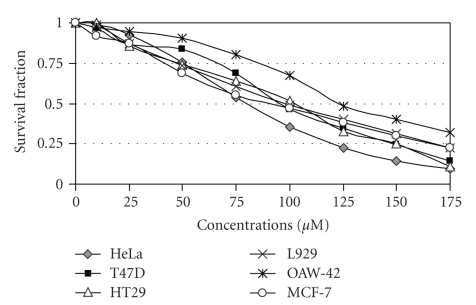
Dose-effect plots of complex **3 **against a panel of human and mouse cancer cell lines 24 h after the administration of the agents. Cytotoxicity was estimated via SRB assay (each point represents a mean of six replicate wells).

Complexes **1 **(Figure 7) and **2** (Figure 8) had no significant inhibition on cellular proliferation against HeLa, HT29 and OAW-42 cancer cell lines and a small effect against L929 normal fibroblastic cell line. In contrast, complex **3** inhibited cellular growth of all cell lines, with IC_50_ concentrations varying between 75 and 125 *μ*M (Figure 9).

**Figure 10:**
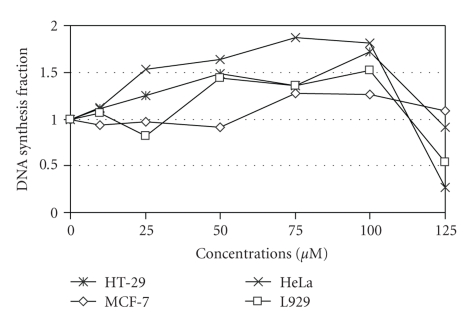
DNA synthesis inhibition of human and mouse cancer cell lines 48 h after the administration of complex **3**.

DNA synthesis was not inhibited in HT29, HeLa, MCF-7 or L929 cell lines when they were exposed to **3** at concentrations up to 100 *μ*M. Higher concentrations exhibited an inhibition of DNA synthesis per cell number only in HeLa and at a lower level in L929 cells (Figure 10).

Treatment with IC_50_ concentrations of **3** for 48fh had no effects on cell cycle distribution of HeLa and T47D cells (Table 5). HT29 and MCF-7 were partially arrested at the G1 phase, OAW-42 were arrested at the G1 phase with a percentage of 87.2% and L929 fibroblasts exhibited a partial G2-phase arrest. However, the overall effect of **3** on cell cycle distribution (except with OAW-42 cells) was not significant, an observation in concert with the results of the BrdU assay, where no inhibition of DNA-synthesis was observed.

## 4. Concluding Comments

In this study, three gallium(III) azole complexes were synthesized and structurally characterized, while their antiproliferative activities were studied. The three different azole ligands were chosen in order to be able to draw structure-properties relations. In two of the complexes (**1** and **2**) the Ga(III) atom is in a trigonal-bipyramidal coordination environment where the terminal azole ligands occupy the axial positions. The third complex (**3**) consists of [GaCl_4_]^−^ anions, chlorine anions and protonated imidazole cations. From the three complexes tested only **3** exhibited a potent anti-proliferative activity against all cell lines tested. The order of cell lines in respect to their sensitivity to **3** (at IC_50_ values) is as follows: HeLa > MCF-7 > T47D > L929 > HT29 > OAW-42. Complex **3** does not inhibit DNA synthesis at concentrations that exert antiproliferative activity (IC_50_s) and does not produce major disturbances in cell cycle distribution (with the exception of OAW-42 cells that, notably, are the most resistant to its anti-proliferative activity).

## 5. Supplementary Information

CCDC 717554, 717555, and 717553 contain the supplementary crystallographic data for **1**, **2**, and **3**. These data can be obtained free of charge from the Cambridge Crystallographic Data Center via http://www.ccdc.cam.ac.uk/data_request/cif.

## Figures and Tables

**Table 1:** Crystallographic data for complexes [GaBr_3_(btaH)_2_] (**1**), [GaCl_3_(btd)_2_] (**2**), and (LH)_2_[GaCl_4_]Cl (**3**).

	**1**	**2**	**3**
Empirical formula	C_12_H_10_N_6_Br_3_Ga	C_12_H_8_N_4_S_2_Cl_3_Ga	C_32_H_30_N_4_Cl_5_Ga
Formula weight	547.68	448.42	717.60
Crystal colour, habit	Colourless, prism	Colourless, prism	Colourless, prism
Crystal dimensions (mm)	0.15 × 0.25 × 0.40	0.20 × 0.25 × 0.40	0.15 × 0.15 × 0.35
Crystal system	Monoclinic	Monoclinic	Triclinic
Space group	*I*2/*a*	*C*2/*c*	*P* − 1
*a* (Å)	16.797(10)	12.098(11)	10.2358(10)
*b* (Å)	7.058(4)	7.525(6)	14.9649(16)
*c* (Å)	14.276(9)	18.968(16)	12.2350(11)
*α* (°)	90	90	69.235(4)
*β* (°)	106.60(2)	107.65(3)	86.879(3)
*γ* (°)	90	90	74.939(4)
*V* (Å^3^)	1621.9(17)	1646(2)	1690.7(3)
*Z*	4	4	2
*D*_calc_ (g/cm^−3^)	2.243	1.810	1.410
*F*(000)	1040	888	732
*μ* (mm^−1^)	9.091	2.411	4.966
Radiation (*λ*, Å)	0.71073	0.71073	1.54180
Temperature (K)	298	298	298
Scan mode	*θ*-2*θ*	*θ*-2*θ*	*θ*-2*θ*
Scan speed (° min^−1^)	3.5	4.2	4.5
Scan range (°)	2.3 + *α*_1_ *α*_2_ separation	2.4 + *α*_1_ *α*_2_ separation	2.25 + *α*_1_ *α*_2_ separation
*θ* range (°)	2.53–25.00	2.25–24.99	3.67–61.97
*hkl* ranges	0 to 19	−14 to 13	−10 to 9
	0 to 8	−8 to 0	−14 to 17
	−16 to 16	0 to 22	0 to 14
Reflections collected	1486	1499	4586
Independent reflections (*R*_int _)	1430 (0.0250)	1450 (0.0219)	4368 (0.0149)
No of refined parameters	121	118	479
Observed reflections [*I* > 2*σ*(*I*)]	1293	1341	3740
*GOF* (on *F* ^2^ )	1.143	1.062	1.087
Final *R* indices^a^[*I* > 2*σ*(*I*)]	*R*_1_ = 0.0332	*R*_1_ = 0.0301	*R*_1_ = 0.0345
	*wR*2 = 0.0903	*wR*2 = 0.0826	*wR*2 = 0.0830
R indices^a^(all data)	*R*_1_ = 0.0375	*R*_1_ = 0.0333	*R*_1_ = 0.0448
	*wR*2 = 0.0930	*wR*2 = 0.0852	*wR*2 = 0.1009
Largest difference peak and hole (e Å^−3^)	0.681 and −1.263	0.546 and −0.387	0.418 and −0.493

^a^Defined as: *R*_1_ = Σ(|*F* *o*| − |*F* *c*|)/Σ(|*F* *o*|), *w* *R*2 = {Σ[*w*(*F*_*o*_^2^−*F*_*c*_^2^)]/Σ[*w*(*F*_*o*_^2^)^2^]}^1/2^, where *w* = 1/[*σ* ^2^(*F*_*o*_^2^) + (*a* *P*)^2^ + (*b* *P*)] with *P* = [max  (*F*_*o*_^2^, 0) + 2*F*_*c*_^2^]/3.

**Table 2:** Selected interatomic distances (*Ǻ*) and angles (°) for complex **1**.

Ga–N(3)′	2.212(3)	N(3)–N(2)	1.314(5)
Ga–N(3)	2.212(3)	N(3)–C(8)	1.378(6)
Ga–Br(2)	2.3204(17)	N(1)–N(2)	1.323(5)
Ga–Br(1)′	2.3436(11)	N(1)–C(9)	1.344(6)
Ga–Br(1)	2.3436(11)	N(1)–HN1	0.89(7)
N(3)′–Ga–N(3)	176.2(2)	Br(2)–Ga–Br(1)′	123.81(3)
N(3)′–Ga–Br(2)	88.08(10)	N(3)′–Ga–Br(1)	90.13(10)
N(3)–Ga–Br(2)	88.08(10)	N(3)–Ga–Br(1)	92.01(10)
N(3)′–Ga–Br(1)′	92.01(10)	Br(2)–Ga–Br(1)	123.81(3)
N(3)–Ga–Br(1)′	90.13(10)	Br(1)′–Ga–Br(1)	112.39(6)

(′)Symmetry code: −*x* + 1/2, *y*, −*z* + 1.

**Table 3:** Selected interatomic distances (*Ǻ*) and angles (°) for complex **2**.

Ga–Cl(1)	2.171(2)	N(3)–C(8)	1.357(4)
Ga–Cl(2)′	2.180(1)	N(3)–S(2)	1.631(3)
Ga–Cl(2)	2.180(1)	S(2)–N(1)	1.601(3)
Ga–N(3)	2.201(3)	N(1)–C(9)	1.336(5)
Ga–N(3)′	2.201(3)		
Cl(1)–Ga–Cl(2)′	120.24(4)	Cl(2)′–Ga–N(3)′	88.34(9)
Cl(1)–Ga–Cl(2)	120.24(4)	Cl(2)–Ga–N(3)′	90.45(9)
Cl(2)′–Ga–Cl(2)	119.52(7)	N(3)–Ga–N(3)′	177.58(12)
Cl(1)–Ga–N(3)	91.21(6)	C(8)–N(3)–S(2)	107.52(19)
Cl(2)′–Ga–N(3)	90.45(9)	C(8)–N(3)–Ga	130.00(19)
Cl(2)–Ga–N(3)	88.34(9)	S(2)–N(3)–Ga	122.41(14)
Cl(1)–Ga–N(3)′	91.21(6)	N(1)–S(2)–N(3)	99.20(15)

(′)Symmetry code: −*x* + 1, *y*, −*z* + 1/2.

**Table 4:** Selected interatomic distances (*Ǻ*) and angles (°) for complex **3**.

Ga–Cl(3)	2.152(1)	C(2)–N(3)	1.309(5)
Ga–Cl(4)	2.166(1)	N(3)–C(4)	1.389(4)
Ga–Cl(1)	2.171(1)	N(11)–C(22)	1.320(5)
Ga–Cl(2)	2.173(1)	N(11)–C(25)	1.392(4)
N(1)–C(2)	1.320(5)	N(11)–C(38)	1.463(4)
N(1)–C(5)	1.393(4)	C(22)–N(13)	1.318(5)
N(1)–C(18)	1.460(4)	N(13)–C(24)	1.383(4)
Cl(3)–Ga–Cl(4)	110.87(5)	Cl(3)–Ga–Cl(2)	110.55(5)
Cl(3)–Ga–Cl(1)	110.17(5)	Cl(4)–Ga–Cl(2)	108.62(5)
Cl(4)–Ga–Cl(1)	109.49(5)	Cl(1)–Ga–Cl(2)	107.05(5)

**Table 5:** Cell cycle distribution of cells before (control) and after 48 h exposure to IC_50_ values of **3** as determined by flow cytometry.

		G1 (%)	S (%)	G2 (%)
HeLa	Control	62.0	26.6	11.4
	**3**	57.7	35.0	7.3
T47D	Control	57.4	28.4	14.3
	**3**	58.1	34.3	7.6
HT29	Control	43.5	39.4	17.1
	**3**	53.2	24.6	22.2
MCF-7	Control	42.0	50.4	7.6
	**3**	54.1	35.4	10.5
OAW-42	Control	47.8	11.3	40.9
	**3**	87.2	11.8	1.0
L929	Control	42.0	48.0	10.0
	**3**	43.0	23.6	33.4
